# Identification of Fluorescent Compounds with Non-Specific Binding Property via High Throughput Live Cell Microscopy

**DOI:** 10.1371/journal.pone.0028802

**Published:** 2012-01-05

**Authors:** Sangeeta Nath, Virginia A. Spencer, Ju Han, Hang Chang, Kai Zhang, Gerald V. Fontenay, Charles Anderson, Joel M. Hyman, Marit Nilsen-Hamilton, Young-Tae Chang, Bahram Parvin

**Affiliations:** 1 Life Sciences Division, Lawrence Berkeley National Laboratory, Berkeley, California, United States of America; 2 Energy Biosciences Institute, University of California, Berkeley, California, United States of America; 3 Department of Biochemistry, Biophysics and Molecular Biology, Iowa State University, Ames, Iowa, United States of America; 4 Department of Chemistry and MedChem Program of Life Sciences Institute, National University of Singapore, and Laboratory of Bioimaging Probe Development, Singapore Bioimaging Consortium, Agency for Science, Technology and Research (A*STAR), Singapore, Republic of Singapore; Virginia Tech, United States of America

## Abstract

**Introduction:**

Compounds exhibiting low non-specific intracellular binding or non-stickiness are concomitant with rapid clearing and in high demand for live-cell imaging assays because they allow for intracellular receptor localization with a high signal/noise ratio. The non-stickiness property is particularly important for imaging intracellular receptors due to the equilibria involved.

**Method:**

Three mammalian cell lines with diverse genetic backgrounds were used to screen a combinatorial fluorescence library via high throughput live cell microscopy for potential ligands with high in- and out-flux properties. The binding properties of ligands identified from the first screen were subsequently validated on plant root hair. A correlative analysis was then performed between each ligand and its corresponding physiochemical and structural properties.

**Results:**

The non-stickiness property of each ligand was quantified as a function of the temporal uptake and retention on a cell-by-cell basis. Our data shows that (i) mammalian systems can serve as a pre-screening tool for complex plant species that are not amenable to high-throughput imaging; (ii) retention and spatial localization of chemical compounds vary within and between each cell line; and (iii) the structural similarities of compounds can infer their non-specific binding properties.

**Conclusion:**

We have validated a protocol for identifying chemical compounds with non-specific binding properties that is testable across diverse species. Further analysis reveals an overlap between the non-stickiness property and the structural similarity of compounds. The net result is a more robust screening assay for identifying desirable ligands that can be used to monitor intracellular localization. Several new applications of the screening protocol and results are also presented.

## Introduction

There is a growing need to identify potential ligands with non-specific binding properties that can easily flow in- and out- of the cells, and compounds with this characteristic provide an enabling step for imaging receptors that are expressed inside the cells. There are several examples of cellular receptors for small molecules for which it is important to know their intracellular or cellular localization through live cell imaging. These receptors include enzymes and proteins that are regulated by small molecules [1], [2], [3], [4]. Currently, the means of tracking intracellular localization of receptors are through immunocytochemistry or the use of fusion proteins such as GFP. The former method cannot be easily used for imaging live cells, while the latter is compromised by the aberrant effect of the fluorescent reporter tag on protein localization or function. This reveals a need for genetic expression of such a labeled protein.

To date, cell surface receptor imaging has successfully been used in animals and cultured cells to localize receptors and determine their specificities. This has been possible because the free receptor ligands can be readily removed from the environment in order to achieve a sufficient signal/noise ratio for imaging. For animals, the blood flow enables the separation of bound and free ligands and the experimentalist can achieve the same in the culture dish by washing the cell surfaces with ligand-free fluids. However, for receptors inside the cell, the cell membrane provides an additional barrier for clearing the free ligands. Consequently, for the imaging of intracellular receptor localization, it is important to have ligands which display low rates of non-specific adsorption to intracellular components, and therefore, maximal rates of removal from a cell (out-flux) when in an unbound state.

The purpose of this study was to identify candidate ligands, which can be used as tags for tracking the intracellular localization of molecules from a library of 240 fluorescent small molecules with R1 and R2 diversities that were synthesized on a scaffold derived from rhodamine, a commonly used fluorescent dye (Figure S1) [5]. We initiated our investigation by screening the compounds in this combinatorial library for their in- and out-flux properties with a quantitative and robust protocol that evaluated the “non-stickiness index” (NSI) per cell with high statistical power. In this study, three genetically diverse mammalian cell lines from human and mouse [6] were initially used as independent biosensors for preliminary screening of the compounds. The adsorption properties of ligands showing the highest NSI measurements were then validated in translucent plant root hairs [7] of Arabidopsis thaliana seedlings [8]. Mammalian lines are amenable to high content screening, and two of them were selected from a panel of human breast cancer cell lines that have been characterized through several genome-wide platforms [6]. The results of this screening assay identify a compound that can potentially be used for monitoring intracellular receptor localization and demonstrate the utility of mammalian systems as pre-screening tools for more complex systems, which are less amenable to high throughput imaging.

We also explore potential associations and overlap between the chemical fingerprint and the non-stickiness property. Accordingly, the SMILES codes for this library were constructed, and physiochemical properties and similarities of the pair-wise molecular structures were computed. The intent is to examine whether chemical fingerprinting can be used as a predictor for in-flux or NSI. Our data provides an atlas of phenotypic responses that will benefit other investigators either for selection of ligands for their uptake properties or NS Index. Below, we will summarize the method and the outcome of our experiment.

## Materials and Methods

### Mammalian cell lines

Primary high content screening was performed on the HS578T [9] and CAMA1 [10] human breast cancer cell lines that are available commercially through ATCC, as well as the mouse mammary ScP2 epithelial cell line [11]. These cell lines were selected for their diversity in molecular signatures, phenotypic responses, and species. HS578T (1.5×10^4^), CAMA1 (2.0×10^4^) and ScP2 (2.0×10^4^) cells were seeded on to the same 96-well plate in 150 ml of phenol red free DMEM (Gibco, NY), which is supplemented with 5% (for ScP2 cells) or 10% (for HS578T and CAMA1) fetal bovine serum. The cells were then allowed to attach and grow for a period between 48 and 72 hrs in a 96-well plate format. Prior to analysis, the growth media was aspirated and replaced with 150 ml of phenol red-free DMEM (Gibco, NY) containing 5 mM of Vybrant DyeCycle Ruby stain (Invitrogen, CA), a live cell nuclear stain. This stain was chosen because it is minimally toxic to mammalian cells and it emits fluorescent signals for DNA content analysis in the near-infrared spectrum, thereby minimizing the potential for photo-bleaching of the fluorescent signal from lower excitation frequencies. Compounds from the fluorescent library were then added to the wells for each cell line to a final concentration of 0.25 nM, and incubated at 37°C for 30 min. Following the uptake period, the cells were washed three times with PBS, and 150 ml of fresh phenol red-free DMEM was added back into each well, then the plates were immediately imaged on a Cellomics ArrayScan imaging system equipped with an XF93 filter. After this initial imaging step, the plates were transferred back to a 37°C incubator for an additional 60 min and then imaged once again. The imaging system was programmed to capture four fields of view per well at excitation frequencies of 655 (near infrared), 549 (TRITC), and 475 (FITC) nm, sequentially. The first step of imaging facilitated the in-flux measurements, while the second imaging step facilitated the out-flux readout. Each experiment consisted of nine plates and the in- and out-flux assays for these plates were performed within 48 hr. Each experiment was performed in triplicate on different dates. All images and their metadata were then transferred to the BioSig imaging bioinformatics system.

### Independent validation and root hair assay

Ligands with a high NSI value (defined as high in- and out-fluxes) in HS578T cells were subsequently tested on the root hairs of *Arabidopsis thaliana* seedlings. In preparation, the Arabidopsis seeds (Columbia ecotype) were first surface sterilized in a solution of 30% bleach + 0.1% SDS in water for 20 minutes, washed four times with water, and suspended in 0.15% agar (in water) at 4°C, in the dark, for at least 2 days. They were then grown on vertical 0.8% agar plates containing 0.5× MS medium (2.2 g/L Murashige and Skoog salts (Caisson Labs), 0.6 g/L MES, and 1% sucrose, pH 5.6) in 120 mmol/square-meter/sec light at 21 degrees (21°) C for 3 days. Following this, seedlings were incubated for 60 min at room temperature in 250 ml liquid 0.5× MS medium containing the candidate test compounds and Vybrant DyeCycle Ruby stain, which were added at the same concentrations used for the primary high content screening. The seedlings were then carefully washed three times in a liquid 0.5× MS medium, transferred to a 6-well chamber glass coverslip containing 250 ml of liquid 0.5× MS medium, and imaged with a Zeiss Axiovert 200 microscope equipped with a Yokogawa spinning disk, Stanford Photonics XR/Mega-10 ICCD and QED InVivo version 3.1.1 software. Seedlings were then incubated for an additional hour and re-imaged under the same image acquisition settings. The exposure times for each compound were identical to those used in the primary high content screening and were kept constant for each replicate experiment.

### Chemical Library

The library was synthesized from two diversities, R1 and R2, through a Grignard reaction (see Supplementary Material S1) with rhodamine as scaffolding. The R1 block varies from “A” to “L,” where the R2 blocks varies from “1” to “33.” Therefore, compounds having diversity “C” will have diversities of “C1” to “C33.” One can hypothesize that compounds having a “C” diversity should have similar functional behaviors. This library is shown in Figure S1.

### Image and quantitative analysis

Three to four fields of views were captured per well. Following background correction, each nucleus within each field of view was segmented for quantifying fluorescent compounds on a cell-by-cell basis. The background correction was performed on an image-by-image basis through automated segmentation of foreground and background using a level-set method [12]. Other policies, such as k-nearest neighbor or modeling the background and foreground as two poisson distributions, provided comparable results. This step was followed by nuclear segmentation, which used the first and second derivative operators [13], in combination with geometric reasoning, to separate potential touching cells [14]. There is abundant literature on nuclear segmentation [15], [16] for fixed samples, but live cell imaging simplifies the problem significantly. When a sample is fixed, it is possible that as a result of DNA leakage, adjacent nuclei can merge; thus, requiring more sophisticated methods for readout on a cell-by-cell basis. The results from the nuclear segmentation step then provided the context for approximating cellular boundaries, which was accomplished through curvilinear tessellation of the regions between cells. As a result, the total fluorescent signal within each tessellated region could be associated with an individual nucleus, hence demonstrating the inherent heterogeneity of the biological system. The compound's response was quantified at two emission frequencies, and the maximum response, from one of the emission frequencies, was retained.

To correct for changes in the positioning of registered nuclei due to a shift in the field of view or the wash cycle, the centroids of segmented nuclei were aligned by estimating an affine transform using least square approximation. Nuclei stain provides a reference for both registration cellular uptake at two different time points. Images and experimental variables were uploaded into an extended version of BioSig [17]. The non-stickiness index was then computed from the total fluorescence of a compound at two different time points for each cell.

The non-stickiness index (NSI) was then measured as the log-ratio of in- and out- fluxes that were computed at two different time points for the same field of view. Due to variability in the emission spectra of the compound library, samples were imaged at both 475 nm and 549 nm, and the wavelength corresponding to the maximum response was used for further measurement. Other variations of the non-stickiness index, such as percentage drop of the fluorescent signal, were also investigated. Though variations in the non-stickiness index could alter the identified top candidates, the log-ratio index is more balanced in terms of outliers that were generated. Let *C_i_* be the *i^th^* cell object; *L_j_* be the *j^th^* compound; and *F(C_i_, L_j_, T_0_)* and *F(C_i_, L_j_, T_1_)* be the fluorescent output at time *T_0_* and *T_1_* respectively. Let *F_max_* = *Max_j_(C_i_,L_j_)* for all cell objects *i* for experiments involving compound *j*. Similarly, let *F_min_ = Min_j_(C_i_,L_j_)*. The non-stickyness index (NSI) is the defined as 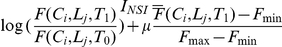, where μ is a constant.

### Chemoinformatics analysis

For each compound, the corresponding SMILE code was generated by computing physiochemical properties and structural dissimilarities between pairwise molecular graphs for chemoinformatics analysis [8], [18]. The structural distances were computed through both SIMCOMP [19], [20] and *JChem* software for comparative analysis. The physiochemical properties were also computed through the JChem software.

Raw data will be annotated and will be made publicly available through BioSig imaging bioinformatics system. In addition, SMILE codes for the combinatorial library will also be published as part of the supplementary materials. Collectively, these data provide the community with an atlas of responses for each cell line, and enables computer scientists with the necessary data to develop alternative methods for structure-activity relationships.

## Results and Discussion

**Figure 1:**
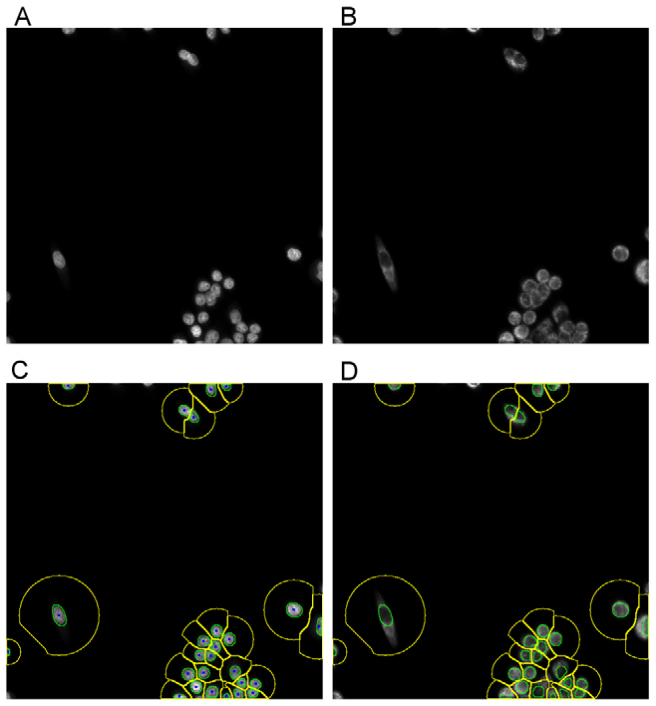
Quantitative analysis and representation of cellular responses. (a) a single field of view from a well with nuclear stain, (b) corresponding compound response, (c) segmentation of nuclei and setting spatial context for readout of the compound response, and (d) previous results overlaid on the compound channel.

**Figure 2:**
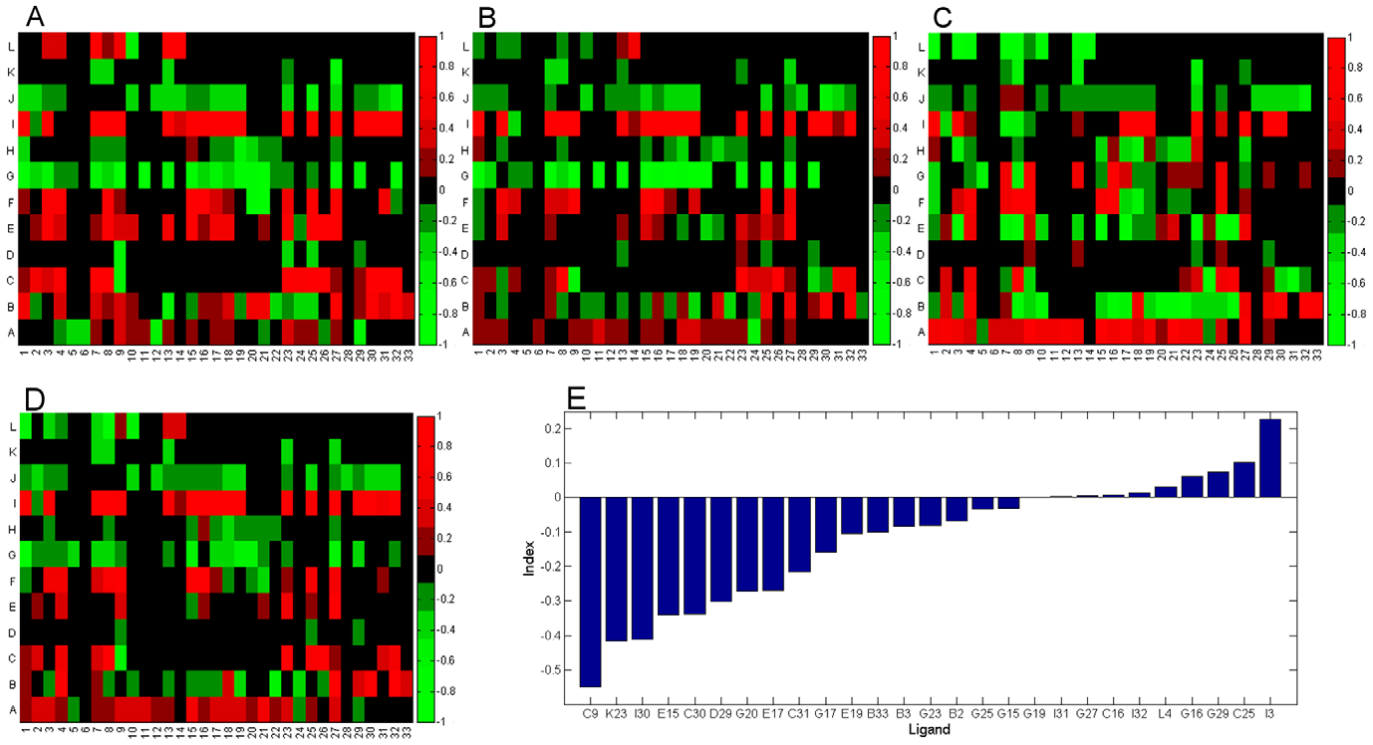
Ranking ligands through integration of heatmap computed from the three cell lines. (a–c) heatmaps for cell lines for CAMA1, HS578T, and ScP2. (d) heatmap for the aggregate of the 3 cell lines, (e) ranked response of compounds on the plant root hair. Each heatmap is represented by an R1 and R2 diversity corresponding to Y and X axis, respectively. The green signal represents a superior non-stickyness index. A subset of compounds (e.g., hits identified by the mammalian lines) is then tested on the plant root hair to reveal that *C9* has a superior response.

In this study, three mammalian cell lines were screened for ligand uptake and retention on a cell-by-cell basis. Each nucleus, from each field of view and each well, was segmented, and the fluorescence in- and out-fluxes were quantified within a nucleus and in a small region surrounding, as show in Figures 1 and S2. Quantified cellular uptake and retentions were subsequently stored in the BioSig system. Initial observations showed that intracellular ligand adsorption and release was inherently heterogeneous within each cell line population. Quantitative analysis of the average NSI values over cells in each field of view was then performed for each cell line. Figure 2 displays heat maps representing the NSI values for each cell line (a–c), as well as the aggregated NSI values over all three cell lines (d), where, in all cases, green and red colors correspond to high and low NSI values, respectively. We have also obtained similar results with other models of aggregation (e.g., nonlinear forms such as median, requiring low NSI in all 3 lines). Ligands with desirable NSI scores (e.g., green blocks that actually have negative values) were then ranked and screened further on the plant root hair to reveal that C9 is the top candidate in our combinatorial library (Figure 2e).

Review and analysis of our experimental data suggests 4 key points of discussion: (I) in- and out-fluxes are cell-line specific, (II) hits from screening mammalian cells can serve as a proxy for plant species, (III) chemoinformatics analysis can contribute to prediction of NSI, though there are always exceptions, and (IV) the combinatorial library used in this study has other potential new applications.

### In- and out-fluxes are cell-line specific

**Figure 3:**
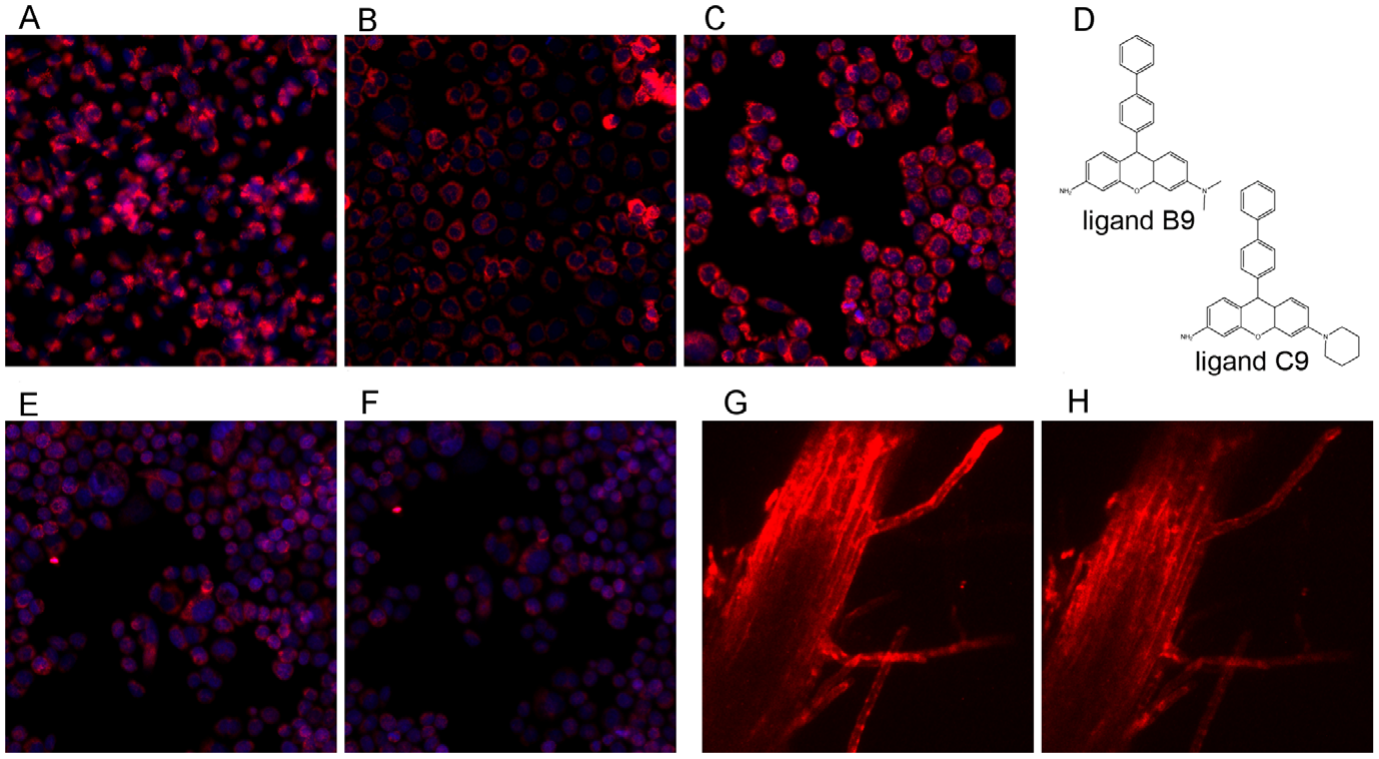
Spatial distribution and non-specific binding signature. (a–c) An example showing that spatial distribution of compound *B9* was heterogeneous for the three mammalian cell lines (a = HS578T, b = Scp2, c = CAMA1 cell lines), (d) structure of *B9* and *C9*, (e–f) images corresponding to in- and out-fluxes for compound *C9* in CAMA1 cell line, (g–h) images corresponding to in- and out-fluxes for compound *C9* in *A. Thailiana* root hair.

Flux studies indicate several phenotypes of interaction between the small fluorescent molecules and cell lines. For example, it may (i) not enter the cell membrane; (ii) bind to the cell membrane and not enter the cell; (iii) enter the cell and accumulate; or (iv) enter and leave the cell over time. These signatures help to classify chemical compound activities into one of several subtypes. Cases (ii) and (iii) refer to sticky compounds, which may have a binding affinity to specific macromolecules, and case (iv) identifies molecules that were selected from our screening process. Interestingly, each cell line had a preferred uptake and retention signature for the chemical library. For example, the mouse cell line, ScP2 [11], tended to support a superior non-stickiness property, while the human cell line, HS578T, showed more retention of the tested compounds (Figure S2). These observations substantiate the need for multiple cell lines when evaluating the NSI properties of potential ligands. With respect to uptake and spatial distribution, again, each cell line had a preferred signature if the compound penetrated the cell membrane. Spatial distribution was either diffused across the entire cell, peri-nuclear, or nuclear-bound. An example of a cell line-specific spatial distribution pattern using compound *B9* (Figure 3d) is shown in Figure 3a–c. In most cases, the uptake patterns in the HS578T and CAMA1 cell lines were peri-nuclear and diffused across the cell, respectively.

### Screening on a mammalian cell line can serve as a proxy for plant species

The non-specific indices aggregated over the three cell lines identified a set of positive and negative hits. The best candidates, along with a few negative hits, were then tested on Arabidopsis root hair. Although C9, shown in Figure 3d, was ranked as the 10^th^ best candidate with uptake and washout shown in Figure 3e–f, respectively, it ranked as the top candidate for the root hair, as shown in Figure 3g–h. This result suggests that correlation across ligands between cell lines and plant root hair may not be very high, but we suggest that mammalian screen can provide a list of candidates. Our data indicates that the *co-occurrence* of a ligand having negative NSI (e.g., desirable non-specific binding) in a mammalian system and plant root hair is relatively high. Correlation and co-occurrence results are shown in the supplementary section in Figure S3 and table S1, respectively.

C9 was able to cross into the nucleus, where it was partially retained following the washout (e.g., outflux). In the root hair, the uptake and retention of *C9* can be interpreted in three ways: (i) this ligand has high in- and out-flux properties, as anticipated, (ii) the uptake of this ligand in the root system is mediated through a cascade of different cell types, or (iii) there is a preferential attachment of compounds at the surface of root hair, which is then washed out, i.e., the compound binds to the cell surface. The first two scenarios are still acceptable for examining intracellular receptors. The last scenario is plausible, but not likely as our time-lapsed studies for the plant system uptake show that the fluorescent compound was sequestered along specific sites in the root structure, and therefore, likely transported by different cell types.

### *Chemoinformatics analysis can be predictive*

**Figure 4:**
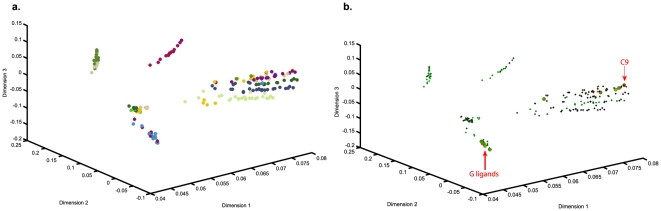
Correlation between NSI values and the chemical structure of the screened ligands. (A) Combinatorial library has 12 diversities corresponding to the R1 block with each diversity painted a distinct color. Therefore, compounds originating from the same diversity should be structurally similar and close to each other in this space, (b) Compounds from (a) with superior NSI properties are shown in large green circles and those that have passed secondary root hair screening process are shown with red outlines.

Several studies were performed to examine correlation of the structural properties of the compounds with both uptake and NSI properties. The association analysis included physiochemical properties and structural distances between pair wise two-dimensional molecular graphs. These properties were directly computed from the *JChem* platform. Our analysis indicates that, although several properties of the physiochemical fingerprints are predictive of uptake (e.g., in-flux), as shown in Figure S4, they are not predictive of the NSI property, shown in Figures S5 and S6. At the same time, visualization through multidimensional scaling (MDS) [21] revealed that structurally similar compounds had similar NSI properties. The *baseline* structural similarities of compounds were also computed and visualized to indicate that the R1 diversity tends to cluster together, as shown in Figure S7. Figure 4 shows the graphical locations of the top 10 most desirable compounds. The color of each dot represents the value of non-specific binding property. Although this method represents an overlap between the chemical and physical response space, it misses C9 as an important target. However, MDS is promising in the sense that some family of ligands can be filtered out. The overlap shown in Figure 4 suggests that screening of many compounds can be bypassed through qualitative inference. As a result, a number of diversities can be eliminated as potential ligand candidates for screening. From this perspective, correlative analysis of the topological structure of compounds provides maps which allow a researcher to decrease the number of compounds in a screening assay. It is interesting to note that many compounds with *G* diversities have high NSI properties, further emphasizing the correlation between the structural attributes and stickiness of a ligand. However, this is not always the case since *C9* was the only compound within its *C* diversity group to display a high NSI value. This is quite intriguing and is a clear indication of how a minor alteration in the topology of a chemical can change its uptake and retention.

### Combinatorial library can inspire novel applications

Figure 2 indicates that each cell line has a unique flux property. In addition, Figure 3 shows that localization properties are also cell-line specific. More precisely, Figure 2 encodes a unique *signature* for a particular cell-line. It is plausible that the uptake or NSI phenotypic signatures of the ligands can be used to identify cells with properties which are specific to cancer cells (e.g., amplification of EGFR). Given that tumors are often heterogeneous and composed of many cell types with different genomic aberrations, one can potentially design an assay to estimate the composition of various cell types in a tumor and subsequently use this information to guide disease treatment on a personalized basis.

Another important application is for developing assays for interrogating uptake or extrusion. Given the baseline heterogeneity of the in- and out-flux properties of the combinatorial library, one can select a compound for its specific flux properties, and conjugate it with a drug to visualize its uptake, translocation or efflux [21] from the cell via membrane proteins. The latter is quite important in intercellular maintenance of cytotoxic drugs and validation of efflux inhibitors as cells acquire multidrug resistance. Compounds with good NSI are particularly suited for developing these assays since drug uptake and efflux can be examined on its own.

### Conclusion

We presented justification for screening for compounds, with a non-stickiness (e.g., non-specific binding) property, which could function as ligands for imaging living cells, and presented a protocol for identifying them from a combinatorial library. The methodology utilizes cell lines from multiple species with diverse molecular signatures for improved robustness. We have shown mammalian cell lines, amenable for high-throughput imaging, can identify hits that can later be tested in three dimensional plant species at a lower throughput, i.e., the mammalian cell lines have the potential to serve as a proxy for other more complex systems. Our protocol is quantitative, and it shows how to make flux measurements on a cell-by-cell basis, as well as how to characterize the NSI. Through dimensionality reduction and visualization, our analysis indicates that a number of compounds, with distinct topological structures, can be excluded from the screening process. Finally, the baseline data provides an atlas of phenotypic responses that will benefit other investigators for developing new assays and new applications.

## Supporting Information

Figure S1 **Combinatorial fluorescent chemical library are synthesized from two diversities of R1 (A–L) and R2 (1–33).**
(EPS)

Figure S2 **Two views of BioSig imaging bioinformatics of the fluorescent compounds indicate that (A) response is heterogeneous for two different cell lines, and (B) the mouse cell line shows excellent non-stickyness property while human cell line does not.**
(EPS)

Figure S3 **Scatter plot of the NSI for compounds in the plant root hair and mammalian cell lines.**
(EPS)

Figure S4 **Correlative analysis of physiochemical properties with cellular uptake reveals small amount of association with the number rotatable bonds and other molecular properties (top row).**
(EPS)

Figure S5 **Correlative analysis of physiochemical properties with the non-stickyness index indicates little correlation.**
(EPS)

Figure S6 **Functional and structural similarities of the top 10 compounds of Figure S5 reveal that (A) while there are similarities of the NSI values between neighboring compounds in** **Figure 2E** **; (B) structural similarities computed through the SIMPCOMP program do not display a similar correlation.**
(EPS)

Figure S7 **Visualization through multidimensional scaling reveals that the R1 diversity is highly correlated structurally, as expected.** Each dot represents a ligand, and the dots with the same color have the same R1 structure.
(EPS)

Table S1 **Co-occurrence distribution of NSI between plant root hair and mammalian cell lines.**
(DOCX)

Supplementary Material S1 **Supporting Information.**
(DOCX)
