# Supplementary material for: Identification of Fluorescent Compounds with Non-Specific Binding Property via High Throughput Live Cell Microscopy
Source: PLoS One. 2012 Jan 5;7(1):e28802. doi: 10.1371/journal.pone.0028802 (PMC3252290; doi:10.1371/journal.pone.0028802)
Supplement: Table S1 — Co-occurrence distribution of NSI between plant root hair and mammalian cell lines. (DOCX) [file pone.0028802.s008.docx]

| Number of ligands | Negative NSI in cell lines | Positive NSI in cell lines |
| --- | --- | --- |
| Negative NSI in root hair | 12 | 5 |
| Positive NSI in root hair | 5 | 4 |
