# Supplementary material for: Identification of Fluorescent Compounds with Non-Specific Binding Property via High Throughput Live Cell Microscopy
Source: PLoS One. 2012 Jan 5;7(1):e28802. doi: 10.1371/journal.pone.0028802 (PMC3252290; doi:10.1371/journal.pone.0028802)
Supplement: Supplementary Material S1 — Supporting Information. (DOCX) [file pone.0028802.s009.docx]

Supplementary Material S1

*Sangeeta Nath, Virginia A. Spencer, Ju Han, Hang Chang, Kai Zhang, Gerald Fontenay, Charles Anderson, Joel Hyman, Marit Nilsen-Hamilton, Young-Tae Chang, and Bahram Parvin*

**1. Combinatorial chemical library**

The library is synthesized from two diversities of R1 and R2 through a Grignard reaction, as shown in Figure S1.

**2. BioSig Imaging Bioinformatics system**

Images and their annotations were uploaded into the BioSig Imaging Bioinformatics system, examples of which, for ligands B2 and E25, are shown in Figures S2A and S2B. For each cell line, the top and bottom rows show the in- and out-fluxes, respectively. These ligands were selected to demonstrate that their spatial distributions can be different for each of the cell lines, or the same cell line at two time points. Figure S2A shows that the distribution pattern is peri-nuclear for HS578T cell line, (ii) and diffused for the mouse ScP2 cell line. Additionally, ligand E25 has excellent flux properties for ScP2 cells, but none for CAMA1. These differential responses can help in the design of a dictionary that can aid in identifying different cell lines and potentially cell types for high throughout put screening of ligand libraries.

**3. Correlation of NSI between mammalian and root hair model system**

We selected a subset of ligands (e.g., Figure 2E) and examined how well non-stickiness indices (NSI) correlate between the plant and mammalian lines. There was a weak correlation across ligands for the two species, as shown in Figure S3. However, given that negative NSI is a desirable property, Table S1 indicates higher co-occurrence of mammalian lines and plan root hair having joint negative NSI. In other words, the mammalian lines can provide a list of candidates for screening in plant species.

**4. Chemoinformatics analysis**

**4.1 Association of physiochemical fingerprints to uptake**

Chemical fingerprints (consisting of 56 features) for each ligand were computed from the corresponding SMILE code through a professional software named *JChem*. The association between each feature and the uptake was studied through a correlation analysis. Figure S4 shows the top 12 chemical attributes that best correlated with cellular uptake. The p-values range from 1.3e-022 to 9.1e-004.

**4.2. Association of physiochemical fingerprints to non-stickiness index (NSI)**

Figure S5 shows the top 12 molecular properties that best correlated to the NSI. The p-values range from 3.9e-9 to 0.06.

**5. Association of structural properties with the top candidate in plant root hair**

The top candidates from the mammalian screen, which revealed the best NSI values were subjected to a secondary screen in the A. Thailiana plant root hair system. The results were shown in Figure 2E in the manuscript. Furthermore, we used the SIMPCOMP program to assess the structural similarities of each compound with one another and then correlated this information with the specific NSI values for each ligand. Again, as in Section 4, structural features were not predictive of the NSI value as shown in Figure S6.

**6. Visualization of structural similarity through multidimensional scaling**

Multidimensional scaling (MDS) was used for visualization of the physiochemical fingerprints for exploring similarities among ligands. MDS is initiated with constructing a similarity matrix between physiochemical or structural properties of the ligands, and then followed by projection in a lower dimensional space, where the original distances are preserved. Figure S7 shows a 3-D visualization of the chemical fingerprints of the ligands through MDS. Here, ligands with the same R1 structure (locations with the same color) are clustered together, which indicates that the similarities of chemical fingerprints are preserved in the low dimensional space.

Figure S1: Combinatorial fluorescent chemical library are synthesized from two diversities of R1 (A-L) and R2 (1-33).

Figure S2: Two views of BioSig imaging bioinformatics of the fluorescent compounds indicate that (A) response is heterogeneous for two different cell lines, and (B) the mouse cell line shows excellent non-specific binding property while human cell line does not.

Figure S3. Scatter plot of the NSI for compounds in the plant root hair and mammalian cell lines.

Figure S4: Correlative analysis of physiochemical properties with cellular uptake reveals a small amount of association with the number of rotatable bonds and other molecular properties (top row).

Figure S5: Correlative analysis of physiochemical properties with NSI indicates little correlation.

Figure S6: Functional and structural similarities of the top 10 compounds of Figure S5 reveal that (A) while there are similarities of the NSI values between neighboring compounds in the bar chart in Figure 2E, (B) structural similarities computed through the SIMPCOMP program do not follow the same profile.

Figure S7: Visualization through multidimensional scaling reveals that the R1 diversity is highly correlated as expected. Each dot represents a ligand, and the dots with the same color have the same R1 structure.

Table S1: Co-occurrence distribution of NSI between plant root hair and mammalian cell lines.

| Number of ligands | Negative NSI in cell lines | Positive NSI in cell lines |
| --- | --- | --- |
| Negative NSI in root hair | 12 | 5 |
| Positive NSI in root hair | 5 | 4 |
